# *Tsc1* deficiency impairs mammary development in mice by suppression of AKT, nuclear ERα, and cell-cycle-driving proteins

**DOI:** 10.1038/srep19587

**Published:** 2016-01-22

**Authors:** Zhenqi Qin, Hang Zheng, Ling Zhou, Yanhua Ou, Bin Huang, Bo Yan, Zhenshu Qin, Cuilan Yang, Yongchun Su, Xiaochun Bai, Jiasong Guo, Jun Lin

**Affiliations:** 1 Department of Histology and Embryology, Southern Medical University, Guangzhou, China; 2 Department of Oncology, Nanfang Hospital, Postal code 510515, Guangzhou, China; 3 Department of Cell Biology, School of Basic Medical Sciences, Southern Medical University, Guangzhou, China; 4 Academy of Orthopedics of Guangdong Province, Department of Orthopedics, The Third Affiliated Hospital, Southern Medical University, Guangzhou, China; 5 Department of Orthopedics, The First People Hospital of Chen Zhou , University of South China, Hunan Province, China; 6 Department of Bioinformatics, School of Basic Medical Science, Southern Medical University, Guangzhou, China; 7 Key Laboratory of Tissue Construction and Detection of Guangdong Province, Guangzhou, China; 8 Institute of Bone Biology, Academy of Orthopedics, Guangzhou,China

## Abstract

Loss of *Tsc1/Tsc2* results in excess cell growth that eventually forms hamartoma in multiple organs. Our study using a mouse model with *Tsc1* conditionally knockout in mammary epithelium showed that *Tsc1* deficiency impaired mammary development. Phosphorylated S6 was up-regulated in *Tsc1*^*−/−*^ mammary epithelium, which could be reversed by rapamycin, suggesting that mTORC1 was hyperactivated in *Tsc1*^*−/−*^ mammary epithelium. The mTORC1 inhibitor rapamycin restored the development of *Tsc1*^*−/−*^ mammary glands whereas suppressed the development of *Tsc1*^*wt/wt*^ mammary glands, indicating that a modest activation of mTORC1 is critical for mammary development. Phosphorylated PDK1 and AKT, nuclear ERα, nuclear IRS-1, SGK3, and cell cycle regulators such as Cyclin D1, Cyclin E, CDK2, CDK4 and their target pRB were all apparently down-regulated in *Tsc1*^*−/−*^ mammary glands, which could be reversed by rapamycin, suggesting that suppression of AKT by hyperactivation of mTORC1, inhibition on nuclear ERα signaling, and down-regulation of cell-cycle-driving proteins play important roles in the retarded mammary development induced by *Tsc1* deletion. This study demonstrated for the first time the *in vivo* role of *Tsc1* in pubertal mammary development of mice, and revealed that loss of *Tsc1* does not necessarily lead to tissue hyperplasia.

The genes *Tsc1* and *Tsc2* got their names from a severe autosomal dominant disorder, called Tuberous sclerosis complex (TSC), resulting from mutations in one or two of these genes. TSC is characterized by formation of hamartomas in multiple organs and tissues, including derma, smooth muscle, kidney and nervous system^1^. Given the serious consequences of mutations in these genes, the functions of *Tsc1* and *Tsc2* have been well investigated. The protein hamartin, encoded by *Tsc1*, and the protein tuberin, encoded by *Tsc2*, form a functional heterodimer (TSC1/TSC2) with a GTPase-activating protein (GAP) activity, which regulates signaling pathways controlling cell size, cell cycle and cellular proliferation^1^,^2^. TSC1 and TSC2 bind to each other via their respective coiled-coil domains^2^. It is TSC2 that contains a C-terminal GAP domain^3^, while binding of TSC1 seems to protect TSC2 against ubiquitin-mediated degradation^4^,^5^. Studies have shown that inactivating mutations in either TSC1 or TSC2 give rise to indistinguishable phenotypes in *Drosophila*. Therefore silence of either of these two genes is equally efficient to inhibit TSC1/TSC2 complex activity^1^.

The mammalian target of rapamycin (mTOR), a serine-threonine kinase belonging to the phosphatidylinositol kinase-related kinase family, is a principal downstream target of TSC1/TSC2 complex^6^. mTOR plays its role in two kinds of protein complexes, mTOR complex 1 (mTORC1)^7^ and mTOR complex 2 (mTORC2)^8^. mTORC1, containing mTOR, regulatory protein of mTOR (Raptor), PRAS40 and mLST8, is directly activated by a small GTPase called Ras-homology enriched in brain (Rheb), whose activation is controlled by TSC1/TSC2 complex^7^,^9^. TSC1/TSC2 complex binds to GTP-bound Rheb and stimulates GTP hydrolysis, so as to inhibit the activity of Rheb and subsequently causes inactivation of mTORC1^10^. Therefore loss of *Tsc1* or *Tsc2* will lead to sustained activation of mTORC1. Activated mTORC1 positively regulates several growth-related cellular process such as transcription and protein translation, which contributes to cell growth and cellular proliferation^11^. Notably, there exist a negative feedback regulation between mTORC1 and AKT, another key protein promoting cell survival and proliferation. When mTORC1 is activated, S6K, one of the mTORC1 downstream target, will disrupt the interaction of insulin receptor substrate-1 (IRS-1) with insulin receptors^12^, leading to a blockage in insulin signaling to AKT^13^,^14^, and consequently suppress the AKT activity. Such negative feedback regulation on AKT by mTORC1 surely plays a vital role in controlling proper cell growth and proliferation.

Tissue and organ development demands appropriate cell growth, proliferation and apoptosis, where mTORC1 signaling exert a critical role. However embryonic lethality caused by knockout of a vital gene such as *Tsc1* hinder our full knowledge about the role of the gene in development. The mammary gland, unlike other organs, undergoes most of its development postnatally with the onset of puberty rather than *in utero*^15^. Here we used a *Cre-LoxP* conditional knockout strategy to build a mouse model with a specific *Tsc1* deletion in mammary epithelium, which can be born and grow into adult ages and allow us to study the *in vivo* effects of *Tsc1* deletion in mammary development. Interestingly, *Tsc1* deficiency did not caused hyperplasia of mammary epithelium but evidently retarded the mammary gland branching by decreasing epithelial cell proliferation and increasing cell apoptosis. This study indicated that a modest mTORC1 activity was critical for pubertal mammary gland development in mice.

## Results

### Conditional knockout of *Tsc1* in mammary epithelium impaired mammary development in mice

**Figure 1:**
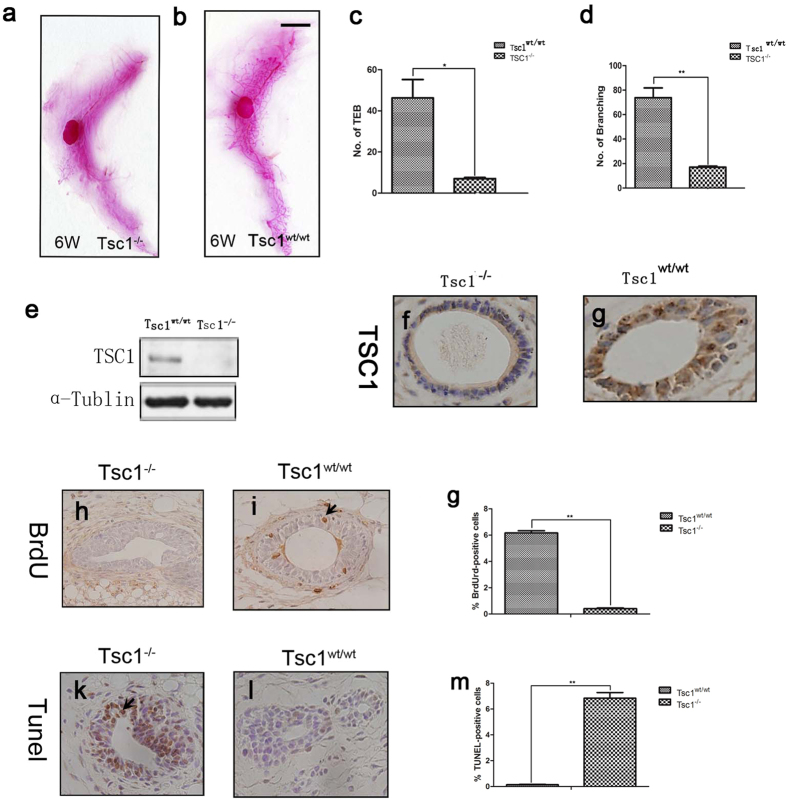
Conditional knockout of *Tsc1* in mammary epithelium led to retarted mammary development in mice. Whole mount staining of the 4th mammary gland of the 6-weeks old female Tsc1^*−/−*^ mice (**a**), *Tsc1*^*wt/w*^ mice (**b**) showed that *Tsc1* deficiency induced less developed mammary gland. The number of TEBs (**c**) and branches (**d**) were compared between *Tsc1*^*−/−*^ mammary glands and *Tsc1*^*wt/wt*^ mammary glands (n = 3). Asterisks indicate the P value (*P < 0.05, **P < 0.01). Expression of TSC1 of mammary glands were detected by immunoblotting (**e**) and immunohistochemistry (**f,g**) to confirm the effect of *Tsc1* deletion. Immunohistochemical detection of BrdU incorporation was performed to *Tsc1*^*−/−*^ mammary glands (**h**) and *Tsc1*^*wt/wt*^ mammary glands (**i**), with comparison of number of BrdU-positive cells of the two genotypes (**j**) (n = 3). Immunohistochemical detection of TUNEL-staining-positive cells was performed to *Tsc1*^*−/−*^ mammary glands (**k**) and *Tsc1*^*wt/wt*^ mammary glands (**l**), with comparison of number of TUNEL-positive cells of the two genotypes (**m**) (n = 3). Asterisks indicate the P value (*P < 0.05, **P < 0.01).

Using a *Cre-LoxP* conditional knockout strategy, we generated a mouse model with a specific knockout of *Tsc1* in mammary epithelium (*Tsc1*^*L/L*^*MMTVCre*^*+*^) to study the role of *Tsc1* in mammary development. At the age of 6 weeks, equivalent to puberty, female *Tsc1*^*L/L*^*MMTVCre*^*+*^ mice (shortened as *Tsc1*^*−/−*^) and *Tsc1*^*wt/wt*^*MMTVCre*^*+*^ mice (the wild type control, shortened as *Tsc1*^*wt/wt*^) were evaluated by whole mount analysis for their mammary glands morphogenesis. It was evident that the mammary ductal tree was less developed in *Tsc1*^*−/−*^ mammary glands than it was in the control *Tsc1*^*wt/wt*^ mammary glands (Fig. 1a,b), confirmed by quantitative comparison of numbers of terminal end buds (TEBs) and mammary branches between *Tsc1*^*−/−*^ and *Tsc1*^*wt/wt*^ mammary glands (Fig. 1c,d). At the same time, immunoblotting and immunohistochemistry was carried out to confirm that *Tsc1* was efficiently knockout in the mammary epithelium of the *Tsc1*^*L/L*^*MMTVCre*^*+*^ mice (Fig. 1e–g). To identify the reasons underlying the poor development of *Tsc1*^*−/−*^ mammary glands, BrdU incorperation staining assays and TUNEL assays were performed to examine the proliferation and apoptotic status of the mammary cells. As is shown in Fig. 1h–m, the number of BrdU-positive cells was markedly decreased while the number of apoptotic cells was increased in the *Tsc1*^*−/−*^ mammary glands, compared with the wild type mammary glands. These data indicated that the pubertal mammary development is impaired in *Tsc1*^*−/−*^ mammary glands.

### Rapamycin restored the mammary development in Tsc1^*−/−*^ mice while suppressed it in Tsc1^*wt/wt*^ mice

**Figure 2:**
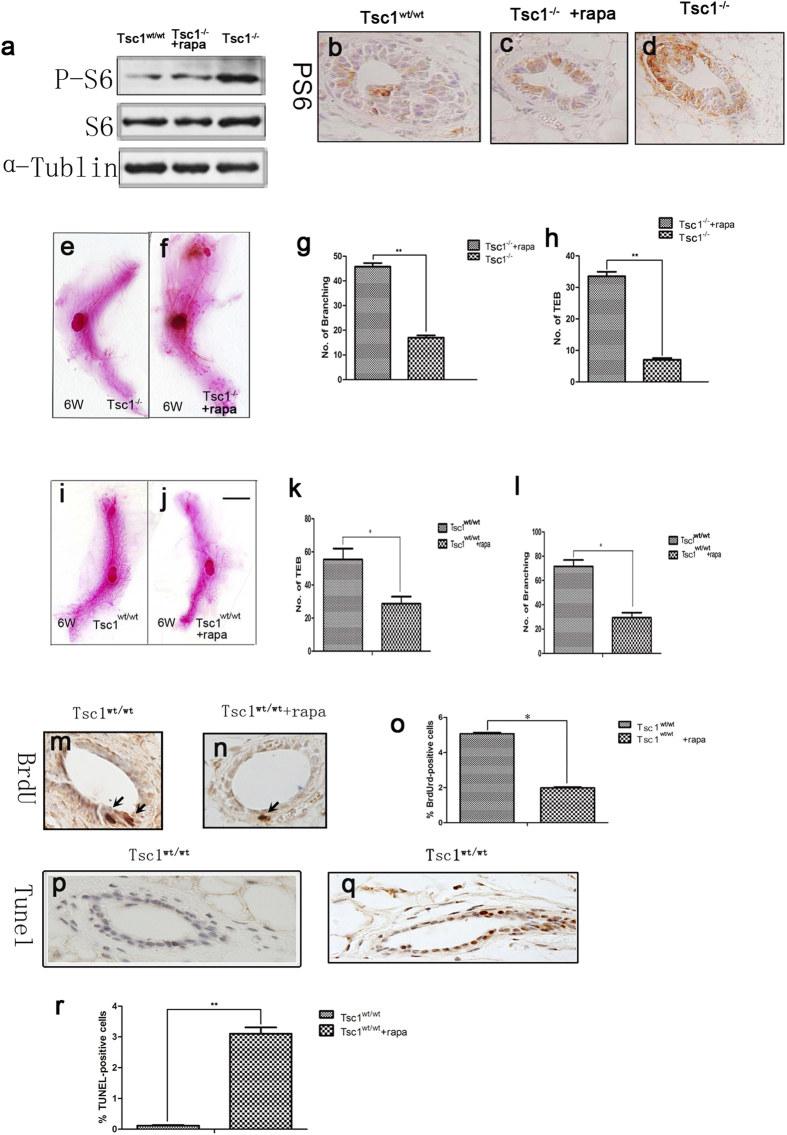
Rapamycin restored the mammary development in Tsc1^*−/−*^ mice while suppressed it in Tsc1^*wt/wt*^ mice. p-S6 was detected by immunoblotting (**a**) and immunohistochemistry (**b–d**) in *Tsc1*^*wt/wt*^ mammary glands, *Tsc1*^*−/−*^ mammary glands and *Tsc1*^*−/−*^ mammary glands treated with rapamycin to showed that mTORC1 was activated by *Tsc1* deletion and could be reversed by rapamycin. Whole mount staining of the mammary glands of female *Tsc1*^*−/−*^ mice treated with saline for 2 weeks and those treated with rapamycin for 2 weeks indicated that rapamycin could rescue the mammary development of Tsc1^*−/−*^ mice (**e,f**). Comparison of the number of TEBs and branches between the two groups were showed in (**g,h**) (n = 3). Asterisks indicate the P value (*P < 0.05, **P < 0.01). Whole mount staining of the mammary glands of female *Tsc1*^*wt/wt*^ mice treated with saline for 2 weeks and those treated with rapamycin for 2 weeks indicated that rapamycin suppressed the mammary development of *Tsc1*^*wt/wt*^ mice (**i,j**). Comparison of the number of TEB and branches between the two groups were showed in (**k,l**) (n = 3). Asterisks indicate the P value (*P < 0.05, **P < 0.01). Immunohistochemical detection of BrdU incorporation was performed in *Tsc1*^*wt/wt*^ mammary glands (**m**) and *Tsc1*^*wt/wt*^ mammary glands treated with rapamycin (n), with comparison of number of BrdU-positive cells of the two groups (**o**) (n = 3). Immunohistochemical detection of TUNEL-staining-positive cells was performed to *Tsc1*^*wt/wt*^ mammary glands (**p**) and *Tsc1*^*wt/wt*^ mammary glands treated with rapamycin (q), with comparison of number of TUNEL-positive cells of the two groups (r) (n = 3). Asterisks indicate the P value (*P < 0.05, **P < 0.01).

Since TSC1/TSC2 complex is an upstream negative regulator of mTORC1, loss of *Tsc1* or *Tsc2* will result in hyperactivation of mTORC1. In the present model, we found that phosphorylation of S6 (p-S6) , a downstream target of mTORC1, was far more enhanced in *Tsc1*^*−/−*^ mammary glands than it was in the *Tsc1*^*wt/wt*^ glands (Fig. 2a–d), indicating that mTORC1 of the mammary epithelium is sustained activated by *Tsc1* knockout. Rapamycin is a specific inhibitor of mTORC1. We then wonder if it could reverse the phenotype observed in the *Tsc1*^*−/−*^ mammary glands. 4-weeks-old *Tsc1*^*L/L*^*MMTVCre*^*+*^ female mice along with their *Tsc1*^*wt/wt*^*MMTVCre*^*+*^ counterparts were treated with rapamycin at a dose of 0.1 mg per kilogram of body weight every other day for 2 weeks. Mice of the same genotype at the same age taking the same dose of saline were taken as control. We found that rapamycin treatment apparently reverse the up-regulation of p-S6 in *Tsc1*^*−/−*^ mammary epithelium (Fig. 2a,d) and rescued the mammary development of these mice (Fig. 2e,f), proved by increased number of TEBs and branches in the rapamycin-treated *Tsc1*^*−/−*^ mammary glands, compared with the saline-treated *Tsc1*^*−/−*^ mammary glands (Fig. 2g,h). However, the same dose of rapamycin repressed the mammary development of female wild type mice (Fig. 2i,j), proved by decreased number of TEBs and branches in rapamycin-treated wild type mice, compared with those of saline-treated wild type mice (Fig. 2k,l). Similar to the *Tsc1*^*L/L*^*MMTVCre*^*+*^ mice, rapamycin-treated wild type mice showed decreased proliferation and increased apoptosis of mammary epithelial cells (Fig. 2m–r). Therefore, a modest activation of mTORC1 plays an important role in normal mammary development. Either sustained activation or inactivation of mTORC1 will impair mammary morphogenesis.

### *Tsc1* knockout undermined the mammary development through suppression of AKT

**Figure 3:**
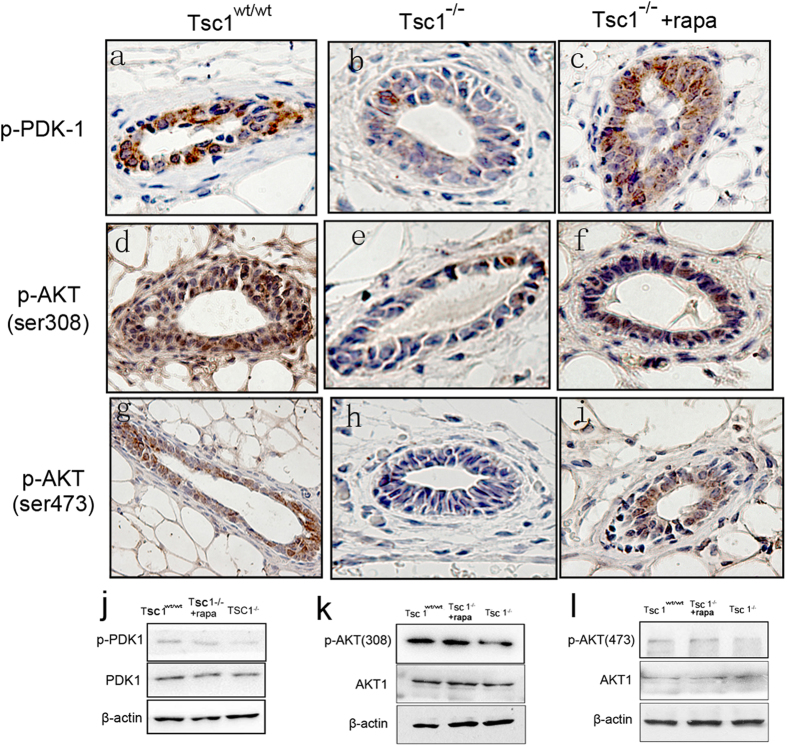
*Tsc1* knockout undermined the mammary development through suppression of AKT. Immunohistochemical detection of phosphorylated PDK-1 (**a–c**), phosphorylated AKT (Ser 308) (**d–f**), and phosphorylated AKT (Ser 473) (**g–i**) were performed in *Tsc1*^*wt/wt*^ mammary glands, *Tsc1*^*−/−*^ mammary glands and *Tsc1*^*−/−*^ mammary glands treated with rapamycin. Immunoblotting detection of these phosphorylated proteins were also performed (**j–l**).

PI3K/PDK-1/Akt pathway plays a critical role in mediating extracellular signals to promote cell proliferation and survival. It is well known that AKT activity can be suppressed by activation of mTOC1 through a negative feedback loop mediated by insulin receptor substrate-1(IRS-1), which serves as an adaptor protein up stream of PI3K/PDK-1^13^,^14^. In this study, phosphorylated PDK-1 (p-PDK-1) and phosphorylated AKT (p-AKT308 and p-AKT473) were all downregulated in *Tsc1*^*−/−*^ mammary glands, which could be rescued by rapamycin treatment (Fig. 3), suggesting that the inhibition on PI3K/PDK-1/AKT was due to sustained activation of mTORC1. Given the important function of AKT in cell proliferation and survival, we believe that suppressed AKT activity underlined the abnormal mammary development induced by *Tsc1* knockout.

### Nuclear ERα and IRS-1 was suppressed in the *Tsc1* ^*−/−*^mammary epithelium

**Figure 4:**
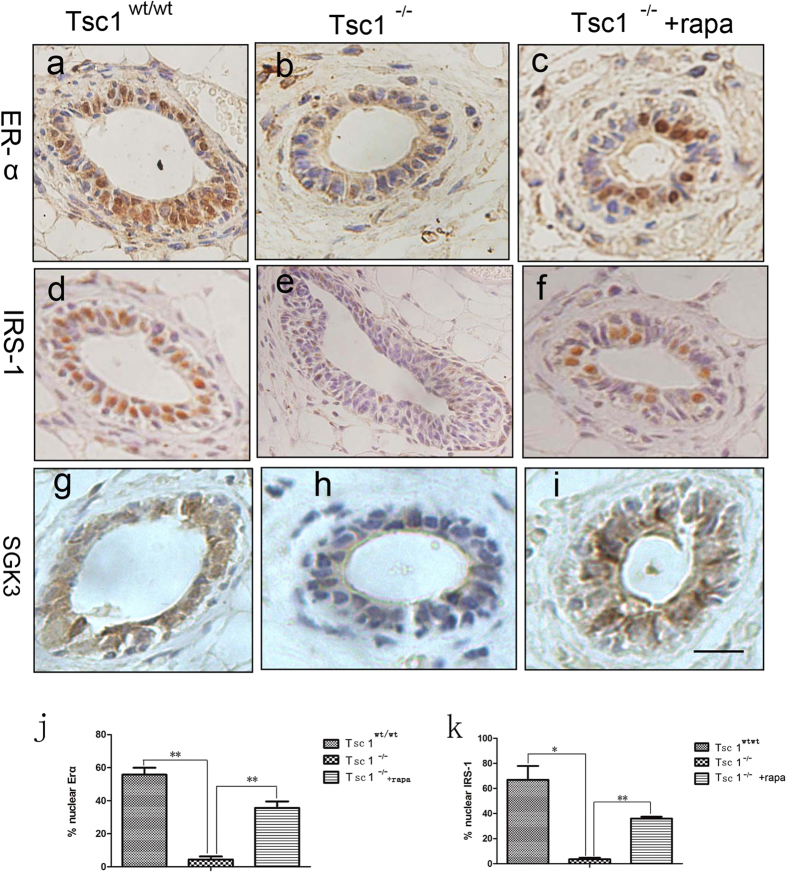
Nuclear ERα and IRS-1 was suppressed in the *Tsc1*^*−/−*^ mammary epithelium. Immunohistochemical detection of ERα (**a–c**), IRS-1 (**d–f**) and SGK3 (**g–i**) were performed in *Tsc1*^*wt/wt*^ mammary glands, *Tsc1*^*−/−*^ mammary glands and rapamycin-treated *Tsc1*^*−/−*^ mammary glands. Quantitative analysis of nuclear-ERα-positive cells and nuclear-IRS-1-positive cells in the 3 kinds of mammary glands were showed in (**j,k**). Asterisks indicate the p value (*P < 0.05, **P < 0.01).

Estrogen is a critical hormone that exerts direct effects on pubertal mammary gland development by stimulating mammary ductal growth^15^. Estrogen receptors (ER) are broadly expressed in both epithelial and stromal compartments of the mammary gland. Upon activation by E2, ERα translocates to the nucleus and functions as a transcriptional activator^15^. IRS-1, the adaptor protein up stream of PI3K/PDK-1, has been demonstrated to be activated by estrogen in breast cancer cells, and then translocates to the nucleus with a direct binding with ERα, indicating a crosstalk between the PI3K/PDK-1/AKT pathway and the ERα pathway^16^,^17^. Our study found out that both nuclear ERα and IRS-1 are down-regulated in *Tsc1*^*−/−*^ mammary glands, whereas rapamycin treatment can resist such down-regulation (Fig. 4a–f). SGK3, an ERα transcriptional target that promotes estrogen-mediated cell survival^18^, was found less expressed in the *Tsc1*^*−/−*^ mammary glands, and was also reversed by rapamycin treatment (Fig. 4g–i). These results implicated that the suppressed nuclear ERα and nuclear IRS-1 might also contribute to the impaired mammary development induced by *Tsc1* knockout.

### Cell cycle related proteins were down-regulated in the *Tsc1* ^*−/−*^ mammary epithelium

**Figure 5:**
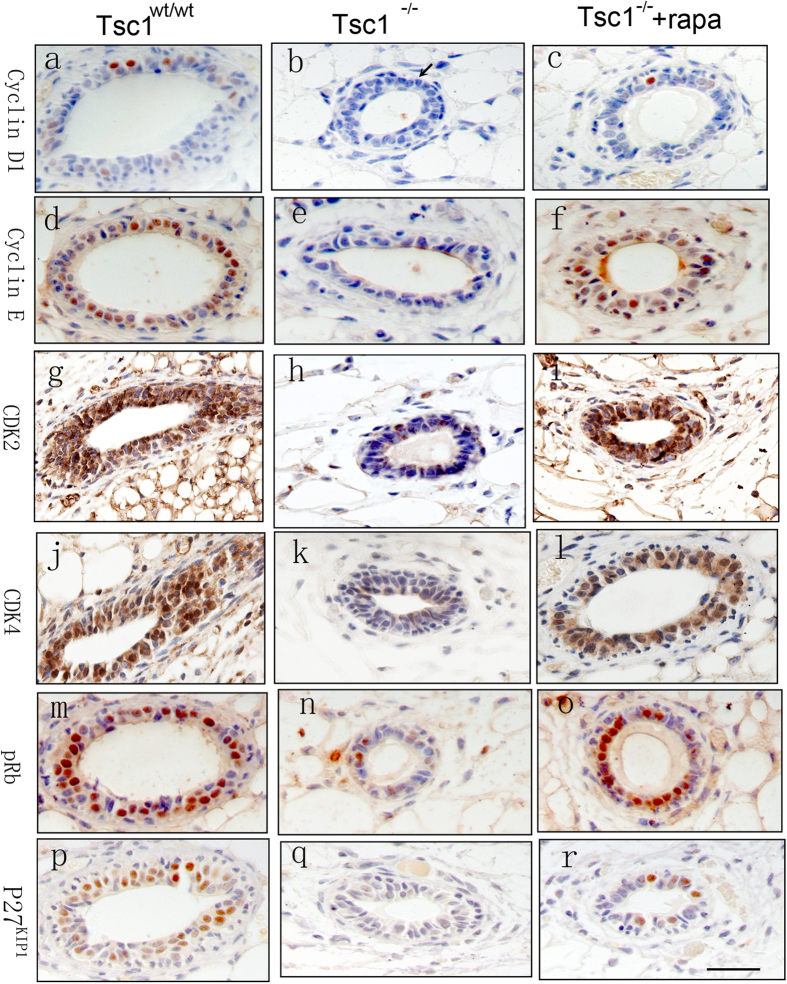
Cell cycle related proteins were down-regulated in the *Tsc1*^*−/−*^ mammary epithelium. Immunohistochemical detection of cyclin D1 (**a–c**), cyclin E (**d–f**), CDK2 (**g–i**), CDK4 (**j–l**), pRB (**m–o**) and p27^kip1^ (**p–r**) were performed in *Tsc1*^*wt/wt*^ mammary glands, *Tsc1*^*−/−*^ mammary glands and rapamycin-treated *Tsc1*^*−/−*^ mammary glands.

According to the present model, loss of *Tsc1* reduced the proliferation of mammary epithelial cells, implying that inhibition of cell cycle might be responsible for the reduced proliferation. The expression of several key proteins regulating cell cycle, such as Cyclin D1, Cyclin E, Cyclin-dependent kinase 2 (Cdk2), Cyclin-dependent kinase 4 (Cdk4), their down stream target pRB and the Cyclin-dependent kinase inhibitor, p27^kip1^, were then investigated in the *Tsc1*^*−/−*^ mammary glands and their wild-type counterparts. It was found that the positive regulators of cell cycle, Cyclin D1, Cyclin E, CDK2 and CDK4, were all down-regulated in *Tsc1*^*−/−*^ mammary glands, and could be rescued by rapamycin treatment (Fig. 5a–i). pRB, phosphorylated by Cyclin D/CDK4 and Cyclin E/CDK2, which results in de-repression of cell cycle progression, was also down-regulated in *Tsc1*^*−/−*^ mammary glands (Fig. 5m–o). Such changes indicated that cell cycle progression was repressed, consistent with the reduced cell proliferation observed in the *Tsc1*^*−/−*^ mammary epithelium. Interestingly, the negative regulator of cyclin/CDK, p27^kip1^ was also down-regulated (Fig. 5p–r), which requires further investigation.

## Discussion

The mammary gland, unlike other organs, undergoes most of its branching postnatally with the onset of puberty rather than *in utero*. Proper branching morphogenesis is fundamental for functional development and differentiation to occur in adulthood^15^. Hormones and growth factors (GH) play a crucial role in mammary ductal morphogenesis. GH signals through GH receptors (GHR) in stromal fibroblasts of mammary, inducing secretion of insulin-like growth factor-1 (IGF-1), which then signals to the mammary epithelium to promote proliferation^19^,^20^. AKT/TSC1/TSC2/mTORC1 is an important pathway transducing the signals from IGF-1 to down stream targets^12^,^13^. However the key components of the pathway, such as TSC1/TSC2 and mTORC1, have not been well investigated in normal mammary development, though their roles in breast cancer development have been known better. Using a mouse model with *Tsc1* specifically deleted in the mammary epithelium, we found that TSC1 is important for mammary development because a modest activation of mTORC1 is needed for proper mammary gland morphogenesis.

Activation of mTORC1 promotes cellular proliferation and growth^7^. TSC1/TSC2 complex exerts negative regulation on mTORC1 signaling. Loss of *Tsc1* or *Tsc2* induces constitutive activation of mTORC1, leading to excessive cell proliferation and cell growth. However unlike the hyperplasia phenotypes of *Tsc1* deficiency that had been observed in other organs such as the kidney^1^, *Tsc1* deficiency in female mammary epithelium caused decreased ductal branching and TEB formation with less BrdU-positive cells and more apoptotic cells, showed by the present study. Such phenotype could be rescued partially by rapamycin, the specific inhibitor of mTORC1, proving that sustained activation of mTORC1 contributes to the abnormal mammary morphogenesis induced by *Tsc1* deficiency. Notably, the same dose of rapamycin repressed the mammary development of female wild type mice (*Tsc1*^*wt/wt*^*MMTVCre*^*+*^), implying that a modest mTORC1 activation is important for normal mammary development, because either sustained activation or inactivation of mTORC1 leads to impaired mammary morphogenesis.

The negative feedback regulation on AKT by activation of mTORC1 may be responsible for the impaired mammary development induced by *Tsc1* deficiency. IRS-1, which binds to insulin receptor or insulin-like growth factor-1 (IGF-1) receptor upon insulin or IGF-1 stimulation, is required for activation of PI3K in response to such stimulus^21^. S6K, once activated by mTORC1, phosphorylates IRS-1 at Ser302, which disrupts the interaction of IRS-1 with the receptors, leading to a blockage in insulin signaling to PDK-1, AKT and mTOR^22^,^23^. The S6K directed phosphorylation of IRS-1 thus constitutes a negative feedback loop that down-regulates the signaling from insulin receptor to AKT. In the present study, phosphorylated PDK-1 and AKT were all apparently down-regulated in *Tsc1*^*−/−*^ mammary glands, which could be rescued by rapamycin treatment, suggesting that the suppressed AKT activity was due to the sustained activation of mTORC1. AKT plays critical roles in cell proliferation and survival^24^. Therefore, inhibition of AKT may contribute to the decreased proliferation and increased apoptosis of mammary epithelial cells. In addition, according to the present model, *Tsc1*^*−/−*^ mice do not develop breast tumors (the longest observation period reached 6 months), which is supposed to be related with the low level of activated AKT.

Estrogen plays a critical role in pubertal mammary development. Through binding to estrogen receptors (ER) broadly expressed in both epithelial and stromal compartments of mammary glands, estrogen stimulates mammary ductal growth by inducing expression of various growth factors^15^. Once activated by estrogen, the ERα is able to translocate into the nucleus and bind to DNA to regulate gene expression, known as the genomic effect of ER. Our study showed that nuclear ERα was down-regulated in the *Tsc1*^*−/−*^ mammary epithelial cells, which could also be reversed by rapamycin, indicating that the genomic effect of ERα was inhibited by sustained activation of mTORC1. The interaction between PI3K/AKT/mTOR signaling and ER signaling has been found in breast cancer cells. Over expression of S6K1, the downstream target of mTORC1, has been reported to regulate ERα by phosphorylating it on serine 167, leading to a transcriptional activation of ERα in breast cancer cells^25^. Suppressed AKT activity influences ER functions in endocrine-resistant breast cancers^26^. But how are these two pathways interact with each other to control normal mammary development needs further investigation. According to the present study, inhibition of nuclear ERα may contribute to the *Tsc1*-deficiency-induced suppressed mammary development. Insulin receptor substrate 1 (IRS-1) is an adaptor protein responsible for transducing signals from insulin, IGF-I, growth factors such as EGF and hormones, including growth hormone and estrogen. Not only functions in cytoplasm, IRS-1 also presents in nuclei and contributes to the process of malignant transformation by activating ribosomal RNA biosynthesis, which stimulates overall protein synthesis^16^,^17^. IRS-1 has been found to directly bind to ERα and co-transfer to the nuclei of breast cancer cells, indicating a interaction between IRS-1 and ERα in controlling breast cancer development^27^. Nuclear IRS-1 was also detected in 1.6% of normal mammary epithelium^28^,^29^. In the present study, nuclear IRS-1 and ERα were all down-regulated in *Tsc1*^*−/−*^ mammary epithelium,which could be reversed by rapamycin, suggesting that their nuclear translocation are all regulated by mTORC1 signaling. The interaction between IRS-1 and ERα may also exist in mammary epithelial cells and contribute to normal mammary development.

The serum- and glucocorticoid-inducible protein kinase (SGK) isoforms are AKT-independent serine/threonine kinases that locate downstream of PI3K/PDK1, playing important roles in PI3K signaling induced cell growth, proliferation and survival independently of AKT^30^,^31^. One of the isoforms, SGK3, can phosphorylate TSC2 and PRAS40, leading to mTORC1 activation^32^. To our current knowledge, the feed back regulation on SGK3 by mTORC1 activation has not been reported. Here we found that SGK3 is down-regulated in the *Tsc1*^*−/−*^ mammary epithelial cells, which can be reversed by rapamycin, implying that a negative feed back regulation may exist between mTORC1 and SGK3. Previous studies also reported that SGK3 is an ERα transcriptional target and promotes estrogen-mediated cell survival of ERα-positive breast cancer cells^33^. Therefore it is also possible that down regulation of SGK3 in *Tsc1*^*−/−*^ mammary epithelia cells is caused by reduced nuclear ERα activity.

Cyclin-CDK complexes play positive roles in driving cell cycle progression^34^. Cyclin D–CDK4 and cyclin E–CDK2 stimulated in G1 phase cause phosphorylation of RB, which release the RB-mediated inhibition of E2Fs and subsequently drive the cell cycle forward to enter S phase^35^. Therefore, the down-regulation of cyclin D1, cyclin E, CDK2 and CDK4 observed in the *Tsc1*^*−/−*^ mammary epithelium could explain why the cell proliferation was inhibited in *Tsc1*^*−/−*^ mammary epithelium. However expression of Cyclin D1 is positively regulated by mTORC1^36^. We speculated that such down-regulation, which could be rescued by rapamycin, might be related to the suppressed AKT activity induced by sustained activation of mTORC1. Consistently, a previous study has reported that Cyclin D1 was less expressed in Akt1-knockout mice mammary glands^37^. p27^kip1^ is a negative regulator of cyclin-CDK complexes including Cyclin D–CDK4 and Cyclin E–CDK2. A direct physical association of p27 with Cyclin–CDK complexes can restrain the CDK activity, thus maintain RB in a hypophosphorylated state that sequesters E2F, which hold back the cell from progression in to S phase^38^. Therefore low expression of p27 is consistent with high cellular proliferation. Multiple human tumors such as breast tumors exhibit abnormally low levels of p27 protein^39^,^40^. Decreased p27 levels in breast tumors correlates with a poor patient prognosis^41^,^42^. However during mammary glands development, the effects of p27^kip1^ on cell cycle seemed to depend on the expression level of p27^kip1^. According to a previous report^43^, *p27*^*+/−*^ mammary glands displayed increased proliferation and delayed involution. But intriguingly, *p27*^*−/−*^ mammary glands displayed a decrease in epithelial proliferation with a marked delay in differentiation, similar to the phenotype of the Cyclin D1–deficient animals. We found that p27^kip1^ was apparently down-regulated in *Tsc1*^*−/−*^ mammary glands, which could be reversed by rapamycin, suggesting that the expression of p27^kip1^ was regulated, at least partially by mTORC1 signaling. Decreased expression of p27^kip1^ was supposed to correlate with increased proliferation, which however has not been observed in the present model. How is p27^kip1^ down-regulated by *Tsc1* deficiency requires further research.

In conclusion, this study supports the notion that a balanced mTORC1 activity was critical for pubertal mammary gland development in mice. Loss of *Tsc1* and consequently sustained activation of mTORC1 results in impaired pubertal mammary gland development. The specific mTORC1 inhibitor rapamycin used at a dose of 0.1 mg per kilogram of body weight every other day for 2 weeks can restore the mammary development of the *Tsc1*^*L/L*^*MMTVCre*^*+*^ mice, whereas delayed the mammary development of the *Tsc1*^*wt/wt*^*MMTVCre*^*+*^ counterparts. Mechanisms underlying the impaired mammary development induced by *Tsc1* deficiency may involve: 1, suppression of AKT by sustained activation of mTORC1; 2, inhibition on nuclear ERα signaling; and 3, down-regulation of Cyclin D1, Cylin E, CDK2 and CDK4, which hold back the cell cycle progression. We speculate that down-regulation of nuclear ERα, Cyclin D1, Cyclin E, CDK2 and CDK4 are related to depressed AKT activity. The negative feed back regulation on AKT by activated mTORC1 probably plays a key role in the retarded mammary development induced by *Tsc1* deficiency.

## Methods

### Animal

Mice harboring the LoxP-flanked *Tsc1* (*Tsc1*^*L/L*^) allele (129S4/SvJae background) were purchased from Jackson Laboratories (Stock No. 005680, Bar Harbor, ME, USA) . MMTVCre^+^ mice were obtained from Center of Model Animal Research at Nanjing University, China. By mating *Tsc1*^*L/L*^ mice with MMTVCre^+^ mice, *Tsc1*^*L/wt*^*MMTVCre*^*+*^ mice were generated, which were then mated with *Tsc1*^*L/L*^ mice to generate *Tsc1*^*L/L*^*MMTVCre*^*+*^ mice with *Tsc1* specifically knockout in mammary epithelia cells. By mating *Tsc1*^*L/wt*^ mice with MMTVCre^+^ mice, *Tsc1*^*wt/wt*^*MMTVCre*^*+*^ mice were generated. All animals were cared for in accordance with guidelines established by the Southern Medical University Animal Care and Use Committee. All experimental protocols were approved by the Southern Medical University Animal Care and Use Committee.

### Rapamycin treatment

The 4-week-old *Tsc1*^*L/L*^*MMTVCre*^*+*^ mice and *Tsc1*^*wt/wt*^*MMTVCre*^*+*^ mice were treated with 0.1 mg/kg rapamycin every other day for 2 weeks by i.p. injections. Counterparts given the same amount of saline were taken as control.

### Whole mount staining, TEBs and branches quantification

The entire 4^th^ mammary gland of each mouse was dissected at 6 weeks of age and spread on a glass slide. Samples were fixed with Carnoy’s fixative for 2-4 hours at room temperature, rinsed in 70% ethanol, and then stained overnight at 4 °C with carmine alum. After dehydration, samples were cleared with xylene and mounted. For TEB quantification, TEBs of greater than or equivalent to 0.03 mm^2^ were counted under a 40 × lens. For branches quantification, the longest primary duct and the secondary duct were counted. Each group took 3 mice to provide the 4^th^ mammary gland, and the TEBs and branches was counted in 3 visual fields per mammary gland under a 40 × lens. The average number of each mammary gland was used for statistical analysis and comparison.

### BrdU incorperation assay and TUNEL assay

Mice at 6 weeks of age were injected i.p. with 10 μl/g body weight bromodeoxyuridine (BrdU) 4 hours before sacrifice. The entire 4^th^ mammary gland of each mouse was removed, fixed in 4% paraformaldehyde for 2-4 h at 4 °C and processed to paraffin block. Sections (5 μm) were cut, dried at 37 °C overnight and then stored at 4 °C for BrdU and TUNEL analyses. To retrieve nuclear antigens on paraffin-embedded sections, slides were incubated for 20 min in sodium citrate buffer (pH 6.0) at 90 °C. The sections were blocked with PGB superblock (10% normal goat serum and 10% BSA in 0.5 m PBS) for 2 h at room temperature and incubated with monoclonal anti-BrdU antibody (1:1500; Millipore) in PGB diluent (PGB superblock with 1% Triton-X-100) in a humidifier box at 4 ° C overnight followed by incubation with fluorescence-conjugated secondary antibodies. To detect apoptotic nuclei, formalin-fixed paraffin-embedded sections were analyzed by TdT digoxigenin nick-end labeling with *in situ* apoptosis detection kit (Promage) following the manufacturer’s instructions.

### Immunohistochemistry

Formalin-fixed paraffin-embedded sections were placed in pressure cooker (full pressure for 3 min) with appropriate 0.01 M sodium citrate (pH 6.0) for antigen retrieval. Subsequently, sections were incubated in blocking solution consisting of 5% BSA and 10% (v/v) normal goat serum in PBS at room temperature for 1 hour. The primary antibodies TSC1((1:100,abclonal), phosphor-S6 (1:100, CST), phospho-PDK1 (1:100, Bioworld), phospho-AKT308 (1:100, Bioworld), phospho-AKT473 (1:100, CST), IRS-1 (1:100, Bioworld), ERα (1:30, Proteintech), cyclin D1 (1:50, Abclonal), cyclin E (1:100, Immunoway), CDK4 (1:100, Abclonal), CDK2 (1:100, Proteintech), p-Rb (1:100, Abclonal) and p27kip1 (1:100, Abclonal) were then applied and incubated at 4 °C overnight. Appropriate secondary antibodies were applied for 1 hour. Nuclei were counterstained with hematoxylin.

### Western Blot

The 4^th^ mammary glands excised from mice were snap frozen in liquid nitrogen, then lysed in cold RIPA buffer (1 × PBS, 1% NP-40, 0.5% sodium dexoycholate, 0.1% SDS, protease inhibitor cocktail tablet [Roche] and 1 mM PMSF [Beyotime Biotechnology]). Lysates were incubated on ice for 20 minutes with frequent vortexing and cleared twice by centrifugation (13,200 rpm,10 minutes, 4 °C). Protein was subjected to SDS-PAGE and transferred onto PVDF membranes (Millipore, Billerica, MA). Membranes were blocked for 60 minutes at room temperature in 5% non-fat milk/Tris-buffered saline/0.1% Tween (TBST). After being washed with TBST, the blots were incubated in primary antibodies for 3 h, which were PS6 (1:2000, CST), S6 (1:2000, CST), TSC1 (1:1000, Proteintech), PDK1(1:1000, ABclonal), phospho-PDK1 (1:1000, Bioworld) phospho-AKT308 (1:1000, Bioworld), phospho-AKT473 (1:1000, CST), AKT1 (1:1000, CST) and β-actin (1:1000, Bioworld). Antibody detection was performed according to the manufacturer’s instructions with ECL Plus Western Blotting Detection System (Genstar, Beijing, China) and developed on film.

### Statistics

Data were analyzed with unpaired, two-tailed Student’s t-test, using a SPSS 10.0 software. The level of significance was set at P < 0.05.

## Additional Information

**How to cite this article**: Qin, Z. *et al*. *Tsc1* deficiency impairs mammary development in mice by suppression of AKT, nuclear ERa, and cell-cycle-driving proteins. *Sci. Rep.* **6**, 19587; doi: 10.1038/srep19587 (2016).
